# Structural Variety and Adsorptive Properties of Mesoporous Silicas with Immobilized Oligosaccharide Groups

**DOI:** 10.1186/s11671-017-2072-2

**Published:** 2017-04-26

**Authors:** Iryna Trofymchuk, Nadiia Roik, Lyudmila Belyakova

**Affiliations:** Chuiko Institute of Surface Chemistry of NAS of Ukraine, 17 General Naumov Str., Kyiv, 03164 Ukraine

**Keywords:** MCM-41, β-cyclodextrin, Sol–gel synthesis, Organosilane, Activating agent, Aromatics

## Abstract

In this research, we report on the synthesis of mesoporous silicas with various quantities of immobilized oligosaccharide groups and different pore ordering degree. The hydrothermal co-condensation of tetraethyl orthosilicate and β-cyclodextrin-containing organosilane in the presence of cetyltrimethylammonium bromide template was employed. The purpose of this investigation was to show the opportunity of increasing β-cyclodextrin content in silica matrix by changing the molar ratio of initial reagents during organosilane synthesis and to determine whether the enhancing of immobilized groups on the surface influences on model aromatic compound adsorption from water. It was prepared several β-cyclodextrin-organosilanes by modification of (3-aminopropyl)triethoxysilane with oligosaccharide (the molar composition of reaction mixtures were 1:1, 3:1, and 5:1) with using *N*,*N*′-carbonyldiimidazole as linking agent. Three types of MCM-41 materials were obtained with 0.018, 0.072, and 0.095 mmol g^−1^ β-cyclodextrin-group loading according to chemical analysis of silicas. The IR spectroscopy and potentiometric titration were also performed to confirm the presence of functional groups in the silica matrix. Nitrogen sorptometry experiments exhibited the decrease of high surface area (from 812 to 457 m^2^ g^−1^) and the average pore diameter (from 1.06 to 0.60 cm^3^ g^−1^) of synthesized silicas with increasing of immobilized oligosaccharide groups. The influence of β-cyclodextrin-organosilane presence on the forming of hexagonally arranged porous structure of silicas was evaluated by X-ray diffraction and TEM analyses. As the loading of oligosaccharide groups increases in obtained silicas, the (100) reflex in diffraction patterns is even less intense and broader, denoting the decrease of long-range pore ordering. Adsorption experiments were carried out to study the effect of β-cyclodextrin groups’ attendance in silica matrix on benzene uptakes from aqueous solutions. Experimental kinetic curves of benzene adsorption on synthesized silicas were compared with theoretical models of Lagergren and Ho-McKay for pseudo-first and pseudo-second-order processes. Langmuir and Freundlich isotherm models were used to evaluate adsorption processes and parameters. Obtained β-cyclodextrin-containing MCM-41 silicas demonstrate adsorption level performance of known samples and could be very promising for benzene uptakes from aqueous solutions in water treatment processes.

## Background

Chemical modification of mesoporous silicas is of great scientific and practical interest since its permit to obtain materials with improved surface and structural properties (large surface areas and ordered mesostructures as well as binding to guest molecules) for potential applications in catalysis, adsorption, drug delivery, and biosensing [1]. Sol–gel synthesis is one of the most widespread methods of producing functional silica, which involves the direct addition of organosilane to silica precursor with mesostructures forming. To obtain stable linkages between silica matrix and functional moieties, the reaction conditions (pH medium, temperature, solvents) as well as functional organosilane compound must be carefully chosen [2]. A variety of chemicals was used as functional organosilanes, like amino-, halo-, sulfido-, methacryloxy-, epoxy-, vinil-, and other alkylsilanes with hydrolyzable methoxy, ethoxy, and acetoxy anchor groups [3–8]. Moreover, to receive desired silylated precursor with specific groups, it is mandatory to use activating or linking agents.

Cyclodextrins are cyclic oligosaccharides composed of several d-glucopyranose units connected by α-(1,4)-linkage, which often applied for the synthesis of functional silica materials. Among them, β-cyclodextrin is one of the most commonly used macromolecules for silica modification. In order to immobilize β-cyclodextrin onto silica, two main approaches were usually applied—postsynthesis modification of silica surfaces with oligosaccharide or its derivatives [9–27] or direct co-condensation of silicon alkoxides with β-cyclodextrin or β-cyclodextrin-containing silanes [28–37]. In the first of these approaches, the modification of β-cyclodextrin with p-toluenesulfonyl chloride to construct β-cyclodextrin derivative as an important intermediate for the preparation of modified silica is often used. For example, thereby nanoporous β-cyclodextrin-containing silicas which differ by functional substituents of wide edge of attached cyclic oligosaccharide molecules (alcohol, bromoacetyl, thiosemicarbazidoacetyl groups) have been synthesized [27]. It was shown that driving force of metal sorption on the surface of functional β-cyclodextrin-containing silicas is the formation of inclusion complexes. In the second, a special attention should be paid on sol–gel synthesis of silicas with immobilized oligosaccharide groups in the presence of different templates [28, 30, 32, 37]. In consequence of this synthesis, it was prepared ordered mesoporous silicas with relatively high surface areas and pore volumes. The microporous cavities provided by covalently bound β-cyclodextrin in these functionalized silica materials are potentially useful as adsorption centers for environmental remediation and chromatographic separations.

The presence of reactive hydroxyl groups in β-cyclodextrin molecule allows attaching oligosaccharides via these groups and an appropriate spacer to the surface. It is obvious that hydrolytically unstable Si–O–C linkages are formed as a result of silanol and β-cyclodextrin hydroxyl groups’ interaction [38, 39]. Hydrolytically stable cyclodextrin-containing silicas could be prepared using oligosaccharide derivatives with appropriate anchor groups. A large number of hydroxyl groups available on two different sides of β-cyclodextrin molecule provide the opportunities of its modification [40]. Methods for selective modification of cyclodextrin can be divided into three categories: the “clever” method (the shortest route), the “long” method (a series of steps to selectively reach the definite position), and the “sledgehammer” method (the desired product separated from isomers and homologues) [41]. Given the fact that modified oligosaccharides are planning to use in sol–gel synthesis processes, one would always choose the most productive and least painful way of chemical transformation. The chemical modification of β-cyclodextrin is carried out using cross-linking or activating agents. Hydroxyl groups of oligosaccharides can be activated with sulfonyl halides, *N*,*N*′-carbonyldiimidazole, succinimidyl chloroformate, epoxides, isocyanates, citric acid, and heterocyclic or alkyl halides [42–46]. Here, *N*,*N*′-carbonyldiimidazole is commonly used reagent for activation in mild conditions. In general, the activation reaction consists of reacting the ones that is modified with an excess of *N*,*N*′-carbonyldiimidazole in anhydrous solvent, such as tetrahydrofuran, chloroform, benzene, dimethylformamide, dichloromethane, and dioxane [43–51].

In our previous study, we used activating ability of *N*,*N*′-carbonyldiimidazole in the reaction with β-cyclodextrin for preparing functionalized MCM-41-type silica materials with hexagonally ordered mesoporous structure [52]. It was exploited two principal methods for β-cyclodextrin-containing MCM-41 silicas producing—postsynthesis attachment to the support by covalent bond formation or sol–gel synthesis using β-cyclodextrin-containing silane in the presence of cetyltrimethylammonium bromide. It was concluded that co-condensation method leads to the formation of β-cyclodextrin-containing MCM-41 silicas with higher arrangement of mesoporous channels compared with one obtained by postsynthesis grafting. Moreover, it was proved that by-products of β-cyclodextrin activation reaction could not affect the structure of the final silica.

This report investigates and compares the structural parameters of β-cyclodextrin-containing MCM-41 silicas with various loadings of oligosaccharide groups and their sorption properties. The co-condensation of tetraethyl orthosilicate and β-cyclodextrin-containing organosilane in the presence of cetyltrimethylammonium bromide template was employed. For templated sol–gel synthesis, three types of β-cyclodextrin-containing organosilanes were prepared by modification of functional silane with oligosaccharide using *N*,*N*′-carbonyldiimidazole as linking agent. The influence of β-cyclodextrin-organosilane presence on the forming of hexagonally arranged porous structure of MCM-41 silicas was evaluated by X-ray diffraction and low-temperature adsorption-desorption of nitrogen. Adsorption experiments were carried out to study the effect of β-cyclodextrin immobilization on silica surface on aromatic compound uptakes from aqueous solutions.

## Methods

### Materials

β-Cyclodextrin hydrate (β-CD) (99%, Acros Organics), tetraethyl orthosilicate (TEOS) (≥99%, Merck), (3-aminopropyl)triethoxysilane (APTES) (≥99%, Merck), *N*,*N*′-carbonyldiimidazole (CDI) (≥98%, Merck), cetyltrimethylammonium bromide (CTMABr) (≥97%, Merck), silver nitrate (pure analytical, Reakhim), and benzene (pure analytical, Reakhim) were used without additional purification. Aqueous ammonia (25%), ethanol (96%), and hydrochloric acid (37%) (Reakhim, all analytical grade) were used as purchased. *N*,*N*′-dimethylformamide (DMF) (pure analytical, Reakhim) was dried for 48 h before utilization with activated molecular sieves (0.3 nm, Merck).

### Synthesis of MCM-41 Silicas

Hexagonally ordered MCM-41 and NH_2_-MCM-41 silicas were prepared by hydrothermal sol–gel synthesis in the presence of structure-directing surfactant, CTMABr, following previously described procedure [53]. In brief, TEOS (or TEOS and APTES mixture) was condensed in alkaline medium with ionic template. Then, hydrothermal treatment of reaction blend in autoclave at 373 K for 24 h was realized. The final molar composition of the reaction mixture for MCM-41 and NH_2_-MCM-41 silicas was as follows: 0.1 TEOS (or 0.09 TEOS:0.006 APTES):0.02 CTMABr:0.55 NH_4_OH:0.56 C_2_H_5_OH:14.4 H_2_O. The template was removed by extraction in acid–ethanol solution.

**Scheme 1:**
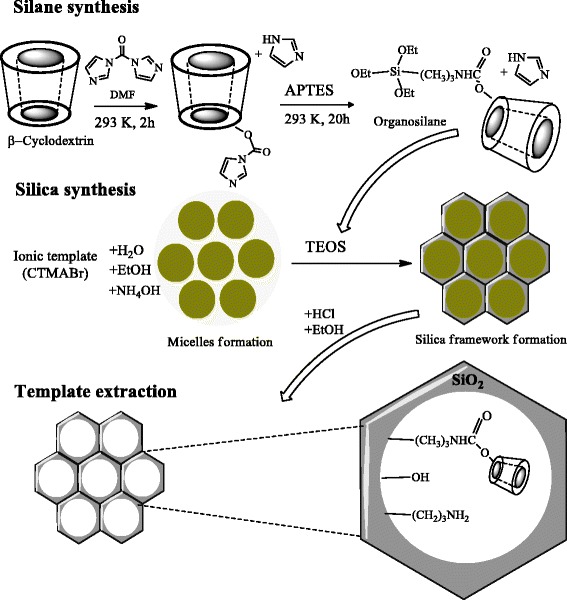
Synthesis of ordered β-cyclodextrin-containing MCM-41 silicas

Synthesis of mesoporous silicas with immobilized oligosaccharide groups comprised three steps (Scheme 1). The first step includes β-CD activation by CDI (molar ratio β-CD:CDI = 1:1, 1:3, or 1:5) in dry DMF with subsequent modification of APTES by aforementioned activated product [22, 52]. The molar composition of reaction mixtures were 1:1, 3:1, and 5:1 for APTES and β-CD, accordingly. The activation reaction was carried out at 293 K for 2 h, while modification of APTES was realized at 293 K for 20 h. As a result, several types of β-CD-organosilanes were obtained. The next step involved the co-condensation of TEOS and a certain type of β-CD-containing organosilane in the presence of CTMABr template. For this, ionic template was dissolved in ethanol-water solution with stirring at room temperature, and then NH_4_OH was added to provide alkaline medium of the reaction. Silica sources (TEOS and β-CD-organosilane product) were added dropwise to reaction mixture under continuous stirring at 293 K. In order to complete the condensation process, the hydrothermal treatment in autoclave at 373 K for 24 h was carried out. The final molar composition of the reaction mixture for silicas preparing was as follows: 0.05 TEOS:0.001 β-CD-organosilane:0.007 CTMABr:0.27 NH_4_OH:7.2 H_2_O. Three MCM-41-type silicas (β-CD-APTES-MCM-41, β-CD-APTES_3_-MCM-41, and β-CD-APTES_5_-MCM-41) were obtained. All silica materials were washed by small quantities of water and dried at ambient temperature. Finally, template was removed by triple solvent extraction in HCl/C_2_H_5_OH solution at room temperature for 24 h. After extraction, silicas were washed with distilled water until the negative test to the halide anions with silver nitrate. Obtained materials were dried in the air at 293 K.

### Characterization

The IR spectroscopy, potentiometric titration, and chemical analysis of surface compounds were performed to confirm the presence of functional groups in the silica matrix. FT-IR spectra of the mesostructures were obtained in transmission mode using Thermo Nicollet NEXUS FT-IR spectrophotometer in the range from 4000 to 400 cm^−1^ for pressed pellets of MCM-41-type silicas. The quantity of aminopropyl groups (${C}_{\left[ N{H}_2\right]}$) on the surface of NH_2_-MCM-41 or β-CD-MCM-41 silicas was calculated by the difference in pH values of starting acid solution (*pH*_*1*_) and equilibrium acid solution with materials batch (*pH*_*2*_) after 24 h contact [54] using Ionometer I-160 by formula:

$$ {C}_{\left[ N{H}_2\right]}=\frac{\left({10}^{- p{H}_1}-{10}^{- p{H}_2}\right) V}{m}, $$

where *V* is the volume of acid solution, L; *m* is the mass of silica, g.

β-CD group loading in the materials was estimated using the acid hydrolysis of oligosaccharide. The concentration of hydrolysis product (glucose) after the reaction with potassium ferricyanide was determined spectrophotometrically at *λ* = 420 nm on Specord M-40 equipment (Germany, Carl Zeiss, Jena) [22, 55].

The ordered mesoporosity of the aminopropyl- and β-CD-containing silicas was confirmed by small-angle (2*θ* = 1–10 grad) X-ray diffraction (XRD) data obtained on a DRON-4-02 diffractometer using CuKα radiation (*λ* = 0.15418 nm) and a nickel filter.

The pore structure of β-CD-containing silicas was observed by transmission electron microscopy (TEM), using a JEM JEOL 1230 electron microscope operated at 100 kV. The formvar film on a Cu grid was used for samples preparation by placing 50 μL of silica suspensions in ethanol on abovementioned support, followed by drying at ambient conditions.

Structural parameters of MCM-41 silicas were also characterized by low-temperature adsorption-desorption of nitrogen. Nitrogen adsorption-desorption isotherms were obtained on Kelvin-1042 Sorptometer for outgassed samples (at 413 K for 20 h). BET specific surface area of MCM-41 silicas was determined from the linear part of adsorption curve in the relative pressure range (*P*/*P*_0_) up to 0.30. The pore size distributions were calculated from isotherm data by applying the NLDFT (equilibrium model). The total pore volume (*V*_total_) was obtained from the amount of nitrogen adsorbed at *P*/*P*_0_ = 0.99.

### Adsorption Study

Pristine MCM-41, amino-functionalized NH_2_-MCM-41, and ordered β-CD-containing (β-CD-APTES-MCM-41, β-CD-APTES_3_-MCM-41, and β-CD-APTES_5_-MCM-41) silicas were applied to the adsorption of aromatic compound from water. The adsorption behavior of benzene in aqueous solution as a function of time and equilibrium concentration was realized by multibatch method at 291 ± 1 K.

For kinetic experiments, air-dried weighted amounts (0.02 g) of each silica and 12 ml of benzene aqueous solution with initial concentration of 0.45 g L^−1^was introduced in air-tight vials. The suspensions were stirred for predetermined time intervals, then the cap of vial was pricked, and solution was collected with syringe and fixed membrane filters on it (pores with *d* = 0.2 μm, PVDF (Millipore)) to prevent the liberation of aromatic compound. The new syringes and filters were used for each sample and experimental point. The content of benzene in filtrates was determined by UV-spectrophotometry at *λ* = 254 nm using standard calibration curves prepared by plotting absorbance at 254 nm of various known concentrations of benzene aqueous solutions (0.01–0.45 g L^–1^). The adsorption amount was calculated according to formula:

$$ {a}_t=\frac{\left({C}_o-{C}_t\right) V}{m}, $$

where *a*_*t*_ is the amount of benzene adsorbed at time *t*, mol g^−1^; *C*_*o*_ and *C*_*t*_ are the initial concentration and the concentration of benzene in filtrate at time *t*, mol L^−1^; *V* is the volume of the aqueous solution of benzene, L; *m* is the mass of adsorbent, g.

Equilibrium adsorption experiments were performed with different initial concentrations of benzene solutions (0.036–0.74 g L^−1^) with the same air-dried weighted amounts (0.01–0.02 g) of each silica in the air-tight vials with 12 ml of investigated mixture. After the adsorption equilibrium was reached, the solution was separated by syringe filter for determination of benzene concentration as in the previous case with kinetic study. The standard calibration curves were used to calculate the initial (*C*_*o*_) and equilibrium (*C*_*eq*_) concentration of benzene solutions from UV absorbance intensity at *λ* = 254 nm. The equilibrium adsorption amount (*a*_*eq*_) was evaluated as:

$$ {a}_{eq}=\frac{\left({C}_o-{C}_{eq}\right) V}{m}. $$

## Results and Discussion

Earlier, a simple surfactant-free route to mesoporous organic–inorganic hybrid silicas containing β-CD groups covalently attached through amide bounds was demonstrated [31]. Surface β-CD “hosts” offer microporous cavities capable for binding “guests” such as organic molecules or inorganic cations. However, these materials have nonperiodic amorphous structures with a low BET surface area from 8 to 386 m^2^ g^−1^. The high specific surface area, thermal and mechanical stability, highly uniform pore distribution, and tunable pores of ordered mesoporous silicas obtained with template sol–gel synthesis may provide greater accessibility of guest molecules to β-CD binding sites. Several attempts were made to introduce β-CD moieties into silica thought co-condensation of silica alkoxides with β-CD-containing organosilanes [28, 30, 32, 33, 37]. In most of these studies, the pore expander was used to obtain the pores of sufficient size. At the same time, it was shown that the pore expander was not completely removed by extraction and its use for the synthesis of such composite materials may cause the presence of entrapped impurities in the formed silica framework [37].

In this research, the new β-CD-containing MCM-41 silicas were prepared using activation ability of CDI by direct co-condensation of β-CD-containing organosilanes and TEOS in the presence of a structure-directing agent without any pore-expanding agent (Scheme 1). The formation of C(O)–N bonds with oligosaccharide molecules under the influence of CDI activator is occurred even at ambient temperature. Elevated temperature (about 373 K) on activation step could lead to the cross-linking of β-CD molecules together instead of activating oligosaccharide fragments for its further cooperation with functional silane [56]. It was confirmed that by-products of β-CD activation reaction could not affect the structure of the final silica, and imidazole can be easily removed from the surface of β-CD-MCM-41 silicas after template extracting step [52]. In addition, the presence of non-toxic imidazole by-product in the reaction mixture during hydrothermal sol–gel synthesis does not lead to drastic changes in pH, and silica structure did not alter [57].

The nature and quantity of functional silane have a significant impact on the formation of the porous structure of the silica matrix. The β-CD-containing hexagonal mesoporous silica synthesis using the “one-pot method” of co-condensation of TEOS, and silylated derivative of β-CD enables to receive materials with high β-CD loadings [28]. However, the used cyanuric chloride linker is sensitive to alkaline hydrolysis, whereas the linker with urethane bond is less susceptible to hydrolysis [37]. Despite the fact that amide linkage is relatively stable, it was shown the opportunity of partial amide bond hydrolysis under hydrothermal treatment in alkaline solution [52]. In order to increase the content of covalently bound β-CD groups on the surface of MCM-41 silicas, it was enhanced the quantities of CDI activator and APTES compound in β-CD-organosilanes synthesis in three and five times for both compounds. Molar ratio β-CD:CDI = 1:1, 1:3, or 1:5 was used, and molar composition of reaction mixtures were 1:1, 3:1, and 5:1 for APTES and activated β-CD, accordingly. In addition, the 10% excess of CDI was used for all β-CD-organosilanes obtaining to ensure complete activation since the purity of CDI is variable due to its extreme sensitivity to water vapor.

**Fig. 1:**
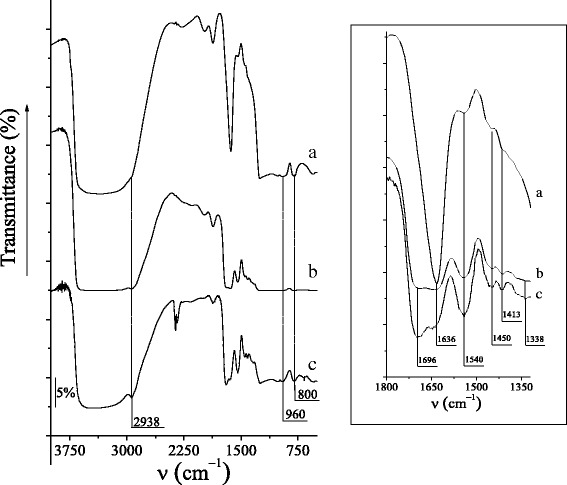
IR spectra of β-CD-APTES-MCM-41 (*a*), β-CD-APTES_3_-MCM-41 (*b*), and β-CD-APTES_5_-MCM-41 (*c*) silicas

Chemical immobilization of cyclic oligosaccharide groups on the surface of silica materials of MCM-41-type was proved by FT-IR spectroscopy (Fig. 1). The silanol groups disposed on the silica surface and remaining water molecules produce the broad stretching band around 3000–3600 cm^−1^, followed by the bands at 1636 and 960 cm^−1^, attributed to the deformation vibrations of the O–H bonds. The strong signal at 1636 cm^−1^ attributed to the deformation vibrations of the O–H bond in the adsorbed water molecules for β-CD-APTES-MCM-41 silica overlapped the possible vibrations of N–H and C=O bonds in this region (inset in Fig. 1, spectrum a). On the contrary, for β-CD-APTES_3_-MCM-41 and β-CD-APTES_5_-MCM-41 silicas, the characteristic absorption bands at 1696 cm^−1^ belonging to the valence vibrations of the C=O bond in the amide linkage are clearly observed (inset in Fig. 1, spectra b and c) [58]. Moreover, the characteristic absorption bands at 1540 cm^−1^ belonging to the deformation vibrations of the N–H bond in the secondary amino groups are registered in the IR spectra of all β-CD-MCM-41 silicas. Also, in the FT-IR spectra of β-CD-APTES-MCM-41, β-CD-APTES_3_-MCM-41, and β-CD-APTES_5_-MCM-41 silicas, the absorption bands at 2938 and 1450, 1413, 1338 cm^−1^ corresponding to the valence and deformation vibrations of the C–H bonds in the alkyl and glycosyl groups of grafted compounds are registered.

The evidence of MCM-41 silicas functionalization was also demonstrated due to chemical analysis of surface compounds. The estimated content of aminopropyl and β-CD groups on the surface of synthesized materials is summarized in Table 1. It was shown that the content of chemically fixed β-CD-containing groups in the silica matrix increases with the number of anchor ethoxysilyl groups in the structure of β-CD-containing silane used in the sol–gel synthesis. β-CD-APTES-MCM-41 silica prepared by modification of APTES and activated oligosaccharide at molar ratio β-CD:CDI = 1:1 exhibit the lowest CD group loading (approximately 0.02 mmol g^−1^). Increasing the amount of CDI (β-CD:CDI = 1:5) at the stage of activation and APTES during silane synthesis results in fivefold growing of immobilized β-CD molecules on the silica surface. The appearance of aminopropyl groups in β-CD-MCM-41 silicas points to the partial hydrolysis of amide bonds under hydrothermal treatment of β-CD-containing silicas in the medium of ammonium. It is evident that proposed sol–gel synthesis leads to bifunctional MCM-41 silica obtaining.

**Table 1:** Structural properties of MCM-41 silicas

Silica	*d*_100_ (nm)	*a* (nm)	*S*_BET_ (m^2^ g^–1^)	*V*_total_ (cm^3^ g^–1^)	*D*_DFT_ (nm)			[−(CH_2_)_3_NH_2_]		[β-CD]	
								(mmol g^−1^)	(μmol m^−2^)	(mmol g^−1^)	(μmol m^−2^)
MCM-41	4.17	4.82	995	0.75	3.7		5.1	−	−	−	−
NH_2_-MCM-41	4.02	4.64	523	0.86	3.7		5.1	0.44	0.84	−	−
β-CD-APTES-MCM-41	4.11	4.75	812	1.06	3.9		5.1	0.05	0.06	0.018	0.022
β-CD-APTES_3_-MCM-41	3.93	4.54	512	0.60	2.5	3.3	5.1	0.11	0.21	0.072	0.141
β-CD-APTES_5_-MCM-41	4.11	4.74	457	0.69	2.4	3.1	4.7	0.12	0.26	0.095	0.208

**Fig. 2:**
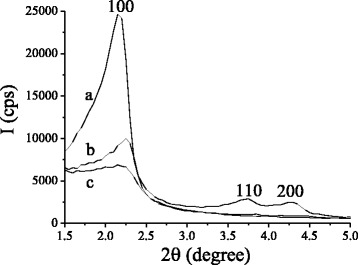
XRD patterns of β-CD-APTES-MCM-41 (*a*), β-CD-APTES_3_-MCM-41 (*b*), and β-CD-APTES_5_-MCM-41 (*c*) silicas

The effect of β-CD group loadings on the forming of hexagonally arranged porous structure of synthesized silicas was evaluated by XRD analysis. XRD patterns of synthesized β-CD-MCM-41 silicas are shown in Fig. 2. The XRD pattern of β-CD-APTES-MCM-41 silica (Fig. 2a) is characterized by the presence of intensive and sharp diffraction peak at 2*θ* = 2.15 grad. attributed to the (100) reticular planes of hexagonally packed pores and confirms the formation of two-dimensionally periodic hexagonal lattice. Moreover, two distinct signals are observed at 2*θ* = ~3.7 and ~4.3 grad. assigned to (110) and (200) reticular planes, which is indication that β-CD-APTES-MCM-41 silica with the lowest β-CD loading has highly ordered mesoporous structure. In comparison with β-CD-APTES-MCM-41, the XRD pattern of β-CD-APTES_3_-MCM-41 and β-CD-APTES_5_-MCM-41 silicas reveals the changes in diffraction peaks. The most intensive (100) reflex for β-CD-APTES_3_-MCM-41 silica (Fig. 2b) is decreased in intensity and slight shifted to high-angle region, evidencing the reduction of interplanar distances *d* in silica framework and the loss of ordering degree. As the loading of β-CD increases in β-CD-APTES_5_-MCM-41 silica, the (100) reflex is even less intense and more broad (Fig. 2c), denoting the variance in structural parameters. In addition, no notable XRD signal attributed to (110) and (200) reticular planes were evident for β-CD-APTES_3_-MCM-41 and β-CD-APTES_5_-MCM-41 materials. Structural parameters (interplanar distance *d* and unit cell parameter *a*) were calculated from the diffraction peak attributed to the (100) reticular planes of synthesized silicas by formulas:

$$ n\lambda \kern0.5em =\kern0.5em 2 d \sin \theta $$

and

$$ a=\frac{2{d}_{100}}{\sqrt{3}}, $$

where *n* is the order of diffraction; *λ* is the wavelength of the X-ray radiation, nm; *θ* is the diffraction angle, grad.

Structural parameters obtained from XRD analysis of previously synthesized hexagonally ordered MCM-41 and NH_2_-MCM-41 silicas [52, 53] and investigated β-CD-MCM-41 silicas are summarized in Table 1. The observed systematic decrease in XRD signal intensity and changes in the values of *d* and *a* parameters upon increasing of the β-CD group loadings is in agreement with other researches, where the use of oligosaccharide moieties during sol–gel synthesis lowers the ordering of materials’ mesoporous structure [28, 30, 32, 37].

**Fig. 3:**
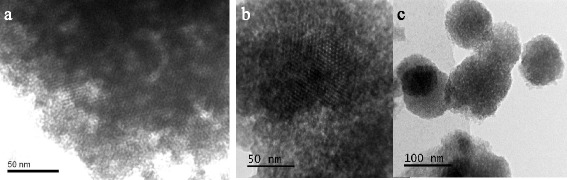
TEM images of β-CD-APTES-MCM-41 (**a**), β-CD-APTES_3_-MCM-41 (**b**), and β-CD-APTES_5_-MCM-41 (**c**) silicas

The pore structure of β-CD-MCM-41 silicas was also investigated by TEM. The microphotographs of the obtained materials are represented in Fig. 3. It should be noted that no notable ordered pore structure of β-CD-APTES_5_-MCM-41 was seen on TEM image (Fig. 3c) in comparison with β-CD-APTES-MCM-41 and β-CD-APTES_3_-MCM-41 (Fig. 3 a, b). As could be clearly seen, the unidimensional cylindrical pores of β-CD-APTES-MCM-41 and β-CD-APTES_3_-MCM-41 silicas are arranged in a honeycomb structure, whereas for β-CD-APTES_5_-MCM-41, the pores are located in disordered manner.

**Fig. 4:**
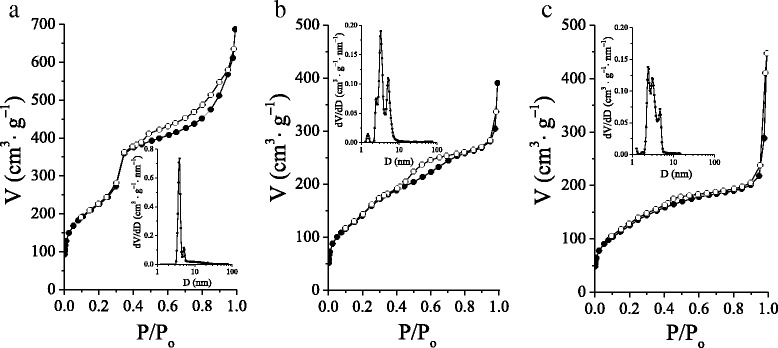
Nitrogen adsorption–desorption isotherms and NLDFT pore diameter of β-CD-APTES-MCM-41 (**a**), β-CD-APTES_3_-MCM-41 (**b**), and β-CD-APTES_5_-MCM-41 (**c**) silicas. *Solid symbols* denoted adsorption, and *open symbols* denoted desorption

The significant changes in porous structure of MCM-41 silicas under the influence of β-CD-organosilane used in templated sol–gel synthesis were also verified by low-temperature adsorption-desorption of nitrogen. The isotherms of nitrogen adsorption-desorption as well as the pore size distributions of synthesized β-CD-MCM-41 silicas are shown in Fig. 4. Structural properties of MCM-41 silicas (BET specific surface areas, total pore volumes, and pore diameters from NLDFT pore size distribution) calculated from nitrogen sorptometry experiments are represented in Table 1. Nitrogen adsorption at low relative pressures (*P*/*P*_0_ <0.3) for β-CD-APTES-MCM-41 silica (Fig. 4a) is attributed to the monolayer formation, following by multilayer adsorption in mesopores. The distinct step on the isotherm at *P*/*P*_0_ ~0.35 indicates a uniformly porous structure. The pore size distribution plot calculated by the NLDFT model clearly demonstrates that uniform pores are prevailing in β-CD-APTES-MCM-41 silica causing the high peak centered at 3.9 nm. The appearance of larger pores (slight peak above 5 nm) can be explained by partial degradation of the walls between individual channels of pores in the process of postsynthesis treatment carried out at 373 K in the medium of ammonia. Hence, the small loading of oligosaccharide groups (nearly 0.02 mmol g^−1^) in β-CD-APTES-MCM-41 silica allows to obtain material with high surface area (812 m^2^ g^−1^), pore volume (1.06 cm^3^ g^−1^), and large-scale sorder porous structure.

The shape of nitrogen adsorption–desorption isotherms for β-CD-APTES_3_-MCM-41 and β-CD-APTES_5_-MCM-41 silicas is slightly different when comparing to material with less β-CD loading (Fig. 4a, b). As can be seen from the isotherms, the nitrogen adsorption at low relative pressures gradually increases, and up to *P*/*P*_0_ ~0.4 only a slight visible inflection step takes place. Such type of isotherms is typical at the sequential formation of adsorbate monolayers on the walls of mesoporous channels with different sizes. Also the distinguished hysteresis loop is observed in the isotherm of β-CD-APTES_3_-MCM-41 silica, characteristic of materials with variable porosity. The pore size distribution plots for these two types of β-MCM-41 materials are wider in comparison with β-CD-APTES-MCM-41, confirming their complex pore structure. For β-CD-APTES_3_-MCM-41 silica, the pore size distribution with three peaks in the region of mesoporous diameters is observed, suggesting the existence of three types of pores with prevailing dominance of 3.3-nm size mesopores (Fig. 4b). Similarly, the broad size distribution is typical for β-CD-APTES_5_-MCM-41 silica with several peaks (at 2.4, 3.1, and 4.7 nm) attributed to mesopores. Furthermore, it is obvious that with increased level of β-CD groups immobilized on the surface of β-CD-APTES_5_-MCM-41 silica, the pore structure becomes less ordered and more tangled. Also, the increased β-CD loading for β-CD-APTES_3_-MCM-41 and β-CD-APTES_5_-MCM-41 silicas in comparison with β-CD-APTES-MCM-41 material leads to the appearance of slight visible peaks on pore size distribution plots in the region of micropore sizes, which is likely to be the result of nitrogen adsorption into the cavities associated with immobilized oligosaccharides on the surface of these silicas. In general, it could be concluded that increased β-CD groups’ attendance in synthesized materials bring out the considerable structural perturbation of the silica framework accompanied by decrease of hexagonal pore ordering. Similar observations were reported in literature [28, 37]. Nonetheless, obtained β-CD-APTES-MCM-41, β-CD-APTES_3_-MCM-41, and β-CD-APTES_5_-MCM-41 silicas have sufficiently high surface areas (457–812 m^2^ g^−1^), pore volumes (0.60–1.06 cm^3^ g^−1^), and β-CD group content (0.018–0.095 mmol g^−1^), which may enhance their adsorption properties in comparison with pristine MCM-41 and aminopropyl functionalized NH_2_-MCM-41 silicas.

Benzene and its derivatives are flammable, toxic, carcinogenic, and/or mutagenic industrial pollutants, which can contaminate the aquatic environment and drinking water because of their high volatility, spreading, and low biodegradability [59]. Among a variety of chemical and physical methods for the elimination of benzene from aqueous solutions, adsorption is one of the most often used and effective approaches. To achieve efficient adsorption of benzene in water, synthesized β-CD-APTES-MCM-41, β-CD-APTES_3_-MCM-41, and β-CD-APTES_5_-MCM-41 silicas were used in this investigation. The benzene adsorption on MCM-41 and NH_2_-MCM-41 silicas were also studied for comparison purpose.

In our previous experiments, it was shown that β-CD-containing MCM-41 silicas exhibit high adsorption ability to benzene molecules, especially at equilibrium concentration of aromatic compound up to 3–4 mmol L^−1^ [52]. The present investigations were carried out to determine whether the increasing of immobilized oligosaccharide groups on the surface enhances the binding abilities of β-CD-containing MCM-41 silicas.

**Fig. 5:**
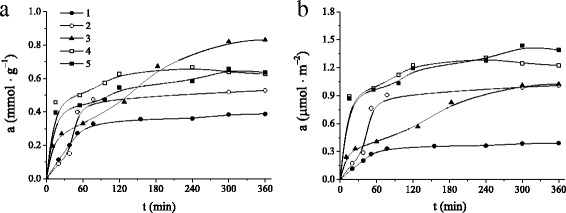
Kinetic curves of benzene adsorption on MCM-41 (*1*), NH_2_-MCM-41 (*2*), β-CD-APTES-MCM-41 (*3*), β-CD-APTES_3_-MCM-41 (*4*), and β-CD-APTES_5_-MCM-41 (5) silicas per gram (**a**) or per square meter (**b**) of materials

In order to estimate equilibrium adsorption time for the uptake of benzene from aqueous solutions by investigated silica materials, time-dependent sorption studies were performed. Kinetic studies show that adsorption of benzene reached saturation for all MCM-41 silicas in less than 5 h. The kinetic curves of benzene adsorption for MCM-41, NH_2_-MCM-41, β-CD-APTES-MCM-41, β-CD-APTES_3_-MCM-41, and β-CD-APTES_5_-MCM-41 silicas are given in Fig. 5. It is evident that biphasic uptake of aromatic molecules with rapid surface bond sorption and long-range diffusion of benzene in pores is typical for most silica adsorbents. The adsorption of benzene molecules on MCM-41, NH_2_-MCM-41, β-CD-APTES_3_-MCM-41, and β-CD-APTES_5_-MCM-41 silicas is very rapid within the first 2 h until it slows down and becomes constant. However, for β-CD-APTES-MCM-41 silica, adsorptive uptake increases gradually to establish equilibrium. Probably, the rate limiting could be due to the difficulty of accessing to adsorbent active sites by the adsorbate.

The pseudo-first and pseudo-second-order kinetic models were used to analyze the adsorption processes on mesoporous silicas for benzene. The linear forms of equations for these models are expressed by Lagergren and Ho-McKay formulas, respectively:

$$ \lg \left({a}_t-{a}_t\right)= \lg {a}_{\mathrm{eq}}-\frac{k_1}{2.303} t, $$

and

$$ \frac{t}{a_t}=\frac{1}{k_2{a}_{\mathrm{eq}}^2}+\frac{1}{a_{\mathrm{eq}}} t $$

where *a*_eq_ and *a*_*t*_ are the amount of benzene adsorbed at equilibrium and at time *t*, mmol g^−1^; *k*_*1*_ and *k*_*2*_ are the rate constants of pseudo-first and pseudo-second-order adsorption processes, min^−1^ and g mmol^−1^ min^−1^, respectively.

The calculated adsorption capacities at equilibrium, rate constants, and regression coefficient values were obtained from the linear plots by fitting the experimental data to both models. These values are listed in Table 2. It has been found that the pseudo-second-order kinetic model provided better correlation coefficients than the pseudo-first-order kinetic model for the adsorption of benzene for all silicas. This assumed that two reactions of rapid reaching of equilibrium and stabilization slow phase were occurred either in sequence or in parallel. In addition, pseudo-second-order kinetic model was confirmed through the better agreement of experimental equilibrium adsorption capacities and calculated values.

**Table 2:** Kinetic parameters of benzene adsorption on MCM-41 silicas

Silica	Pseudo-first-order kinetic model (Lagergren)			Pseudo-second-order kinetic model (Ho-McKay)		
	*k*_1_ (1 g^−1^)	*a*_eq_ (mmol g^−1^)	*R* ^2^	*k*_2_ (g mmol^−1^ min^−1^)	*a*_eq_ (mmol g^−1^)	*R* ^2^
MCM-41	0.013	0.261	0.948	0.060	0.431	0.997
NH_2_-MCM-41	0.013	0.344	0.920	0.018	0.663	0.961
β-CD-APTES-MCM-41	0.014	1.067	0.944	0.011	1.016	0.964
β-CD-APTES_3_-MCM-41	0.010	0.142	0.806	0.159	0.658	0.998
β-CD-APTES_5_-MCM-41	0.009	0.340	0.974	0.052	0.685	0.995

**Fig. 6:**
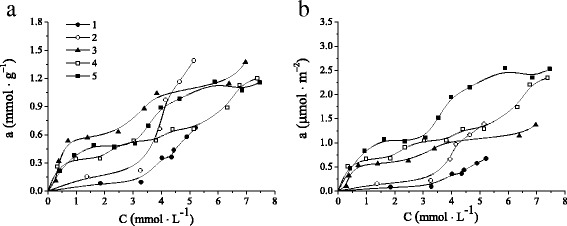
Benzene adsorption isotherms on MCM-41 (*1*), NH_2_-MCM-41 (*2*), β-CD-APTES-MCM-41 (*3*), β-CD-APTES_3_-MCM-41 (*4*), and β-CD-APTES_5_-MCM-41 (*5*) silicas per gram (**a**) or per square meter (**b**) of materials

The existence of functional β-CD groups on the surface of investigated materials causes not only the structural changes but also affects on the sorption behavior of synthesized silicas. The adsorption ability of MCM-41, NH_2_-MCM-41, β-CD-APTES-MCM-41, β-CD-APTES_3_-MCM-41, and β-CD-APTES_5_-MCM-41 silicas was characterized in terms of its benzene adsorption isotherms from aqueous solutions (Fig. 6). A close inspection of adsorption isotherms at low solute equilibrium concentrations (up to 3 mmol L^−1^) reveals that the benzene uptake by MCM-41 or NH_2_-MCM-41 and β-CD-containing silicas differs significantly. As can be seen from the Fig. 6a, where the adsorption isotherms are given as quantity of benzene adsorbed from water per unit weight of silica, the uptake of aromatics on CD-containing silicas increases in comparison with silicas without supramolecular moieties in the region of discussed concentrations. It can be seen that adsorption isotherms of MCM-41 and NH_2_-MCM-41 silicas have concave shape in the region of small equilibrium concentrations of adsorbate as a result of weak affinity of non-polar aromatic rings to polar silanol groups. In contrast, the equilibrium adsorption amount of benzene rises sharp with an increase in the equilibrium concentrations up to 3 mmol L^−1^ for β-CD-APTES-MCM-41, β-CD-APTES_3_-MCM-41, and β-CD-APTES_5_-MCM-41 silicas. Apparently, the higher uptake of benzene is caused by non-specific binding interaction of π-electrons of the aromatic ring with the surface silanol groups [60] as well as selective binding sites within its structure. It was shown that β-CD and benzene could form “host–guest” inclusion complexes in aqueous solutions. The formation of “β-CD–benzene” complexes is spontaneous and thermodynamically profitable exothermal process [61, 62]. However, the clear relation on the number of immobilized oligosaccharide groups and adsorption capacities of β-CD-MCM-41 silicas have not been observed. Moreover, when the equilibrium concentration of benzene is greater than approximately 3 mmol L^−1^, a sharp increase of aromatics uptake for MCM-41 and NH_2_-MCM-41 silicas is registered. Obviously, it could be explained by the reorientation of benzene molecules owing to increase of its concentration in solution, and the more benzene is already adsorbed, the easier it is for additional amounts to become fixed as a result of hydrophobic interactions [63]. The increasing in adsorption capacity of NH_2_-MCM-41 silica toward benzene could be explained by intensifying hydrophobic interaction between aromatic molecules and carbon chains of aminopropyl fragments.

However, taking into account the differences in silica surface areas, the positive effect of β-CD loading on the surface of synthesized adsorbents is evident. The adsorption isotherms of benzene from aqueous solutions on MCM-41, NH_2_-MCM-41, β-CD-APTES-MCM-41, β-CD-APTES_3_-MCM-41, and β-CD-APTES_5_-MCM-41 silicas as quantity of aromatics adsorbed per unit of adsorbent surface are shown in Fig. 6b. Obviously, as the quantity of immobilized β-CD groups increases, the adsorption ability in a series of adsorbents β-CD-APTES-MCM-41 < β-CD-APTES_3_-MCM-41 < β-CD-APTES_5_-MCM-41 raises in the region of small equilibrium concentrations.

The equilibrium adsorption isotherms of benzene on synthesized silicas were analyzed by use of Langmuir and Freundlich models of adsorption. It is known that Langmuir model based on the assumption that adsorption takes place at specific uniformly distributed adsorption sites without significant interaction among adsorbed molecules. Therefore, this model is often used to evaluate monolayer adsorption at homogenous sites, while Freundlich model is applicable to both monolayer and multilayer adsorption on heterogeneous surface. A linear forms of the Langmuir and Freundlich equations, respectively, were used to determine isotherms parameters:

$$ \frac{C_{\mathrm{eq}}}{a_{\mathrm{eq}}}=\frac{1}{a_m{K}_L}+\frac{C_{\mathrm{eq}}}{a_m} $$

and

$$ \lg {a}_{\mathrm{eq}}= \lg {K}_F+\frac{1}{n} \lg {C}_{\mathrm{eq}}, $$

where *C*_eq_ is the equilibrium concentration of adsorptive in a solution (mmol L^−1^); *a﻿*_*eq*_ is the equilibrium adsorption (mmol g^−1^); *K*_*L*_ is the Langmuir constant that characterizes the adsorption energy (L^−1^ mmol); *a*_*m*_ is the adsorption capacity of monolayer (mmol g^−1^); *K*_*F*_ is the Freundlich constant (L^−1^ mmol); 1/*n* is the Freundlich constant characteristic of adsorption intensity. The calculated parameters from both models are summarized in Table 3. The obtained results indicate that the Freundlich isotherm was more suitable for expressing the adsorption of benzene on synthesized silicas. In the case of benzene adsorption on MCM-41 and NH_2_-MCM-41 silicas, correlation coefficients of Langmuir plots are low (${R}_L^2 = 0.711$ and ${R}_L^2 = 0.586$, respectively) to ascribe the occurring adsorption process. At the same time, the results of the adsorption isotherm on β-CD-APTES-MCM-41 reveal that ${R}_L^2$ and ${R}_F^2$ values of linear plots were higher than 0.90, implying that the adsorptions of benzene evaluated by both the Langmuir and Freundlich models. It is also evident that Freundlich model yields a considerably better than Langmuir ones for benzene adsorption on β-CD-APTES_3_-MCM-41 and β-CD-APTES_5_-MCM-41 materials.

**Table 3:** Parameters of Langmuir and Freundlich isotherm models for benzene adsorption on MCM-41 silicas

Silica	Langmuir model			Freundlich model		
	*K*_L_ (L mmol^−1^)	*a*_m_ (mmol g^−1^)	${R}_L^2$	*K*_F_ (L g^−1^)	*n*	${R}_F^2$
MCM-41	-0.154	-0.165	0.711	0.017	0.468	0.901
NH_2_-MCM-41	-0.135	-0.525	0.586	0.071	0.594	0.880
β-CD-APTES-MCM-41	0.334	1.780	0.920	0.426	1.643	0.925
β-CD-APTES_3_-MCM-41	0.192	1.638	0.752	0.345	2.088	0.917
β-CD-APTES_5_-MCM-41	0.205	1.858	0.837	0.352	1.698	0.965

Benzene and its derivatives are frequently used as model compounds of water contaminants to investigate the removal of toxic chemicals from liquid solutions onto different types of adsorbent. It was shown that typical adsorption capacities toward benzene of organo-minerals are 1.257 mg g^−1^ for natural clays, 27 and 28 mg g^−1^ for natural zeolites and montmorillonites, 40 and 365 mg g^−1^ for activated carbon from natural material [64–66]. The adsorption capacities of synthetic materials are 15 and 150 mg g^−1^ for zeolites [67, 68], 36–248 mg g^−1^ for carbon nanotubes [69, 70], 66–274 mg g^−1^ for activated carbon [71–74], and 7 or 100–185 for silica materials [67, 75, 76]. Among these adsorbents, carbon materials are the most popular employed in the adsorption process because of large specific surface area and high capacity. However, these adsorbents also have inherent limitations such as poor mechanical strength and difficult regeneration. At the same time, silica is material with unique properties, like physical strength and chemical inertness that affords to obtain different composites with carbon-based compounds through its modification.

From the adsorption study, it is evidently that prepared β-CD-containing MCM-41 silicas demonstrate adsorption level performance (adsorption capacities around 100 mg g^−1^) of known samples. These easy-to-prepare and reusable materials expect to be the promising for the treatment of aqueous solutions not only from benzene but also from its derivatives. For the last years, it was shown high adsorption efficiency of β-CD-containing adsorbents in the absorption of different aromatic compounds from water [14, 18, 24–26, 29, 35, 36, 45, 46]. Nowadays, there is an increasing interest in the research and development of adsorbent with satisfying adsorption capacity, high mechanical strength, high reusability, and ready availability, especially the adsorbent based on natural products for their ready availability and excellent biocompatibility, which will not cause additional environmental pollution in their application [26, 45, 46]. Particularly interesting is the study, where the natural pine dust was modified with citric acid and β-CD for aniline removal from aqueous solutions [46]. In this case, the carboxyl sites and hydrophobic cavity generated by grafted β-CD improved the hydrophobic property of the adsorbent and the selectivity for aniline. The adsorption capacity of this biosorbent is significantly higher than that of other low-cost adsorbent (kaolin, molecular sieves, and polymers).

Finally, immobilization of cyclic oligosaccharides onto solid supports like MCM-41 silicas makes possible an efficient removing of aromatic pollutants from aqueous solutions by means of supramolecular structures formation, especially at low levels of aromatic compounds. At the same time, it should be noted that the general concept “the more β-CD are immobilized, the more adsorption is observed” may not be always apply to β-CD-MCM-41 materials due to the changes in silica structure (decrease of hexagonal pore ordering, surface area and pore volume) as a result of synthetic conditions [37].

## Conclusions

In this research, structural variety and adsorptive properties of mesoporous silicas with immobilized oligosaccharide groups were investigated. It was realized hydrothermal sol–gel synthesis of three β-CD-containing MCM-41-type silicas with different concentration of functional groups. β-CD-containing silane and tetraethyl orthosilicate as silica sources were applied for co-condensation in the presence of ionic template. Several types of β-CD-silanes were prepared by modification of APTES with oligosaccharide activated by *N*,*N*′-carbonyldiimidazole at various molar ratio of reaction mixture. Obtained functional materials were characterized by FT-IR spectroscopy, chemical and XRD analysis as well as low-temperature adsorption–desorption of nitrogen. It was shown that increased β-CD groups’ attendance in synthesized materials bring out the considerable structural perturbation of the silica framework accompanied by decrease of hexagonal pore ordering, surface area, and pore volume. However, the presence of more immobilized oligosaccharide groups on the silica surface increases the adsorption ability of synthesized materials in the region of small equilibrium concentrations. This is evidence that immobilized β-cyclodextrins’ functional groups have a higher affinity for benzene adsorption as compared with other surface centers. The proposed synthesis route may be useful for obtaining of β-CD-containing MCM-41 silicas with high affinity to aromatic compounds of suitable geometry for water treatment processes using.

## Abbreviations

APTES: (3-Aminopropyl)triethoxysilane
BET: Brunauer–Emmett–Teller
CDI: *N*,*N*′-Carbonyldiimidazole
CTMABr: Cetyltrimethylammonium bromide
DMF: *N*,*N*′-Dimethylformamide
NLDFT: Nonlocalized density functional theory
PVDF: Polyvinylidene fluoride
TEM: Transmission electron microscopy
TEOS: Tetraethyl orthosilicate
XRD: X-ray diffraction
β-CD: β-Cyclodextrin
